# Clinical Heterogeneity in *MT-ATP6* Pathogenic Variants: Same Genotype—Different Onset

**DOI:** 10.3390/cells11030489

**Published:** 2022-01-30

**Authors:** Sara Capiau, Joél Smet, Boel De Paepe, Yilmaz Yildiz, Mutluay Arslan, Olivier Stevens, Maxime Verschoore, Hedwig Stepman, Sara Seneca, Arnaud Vanlander

**Affiliations:** 1 Department of Laboratory Medicine, Ghent University Hospital, 9000 Ghent, Belgium; hedwig.stepman@uzgent.be; 2 Department of Child Neurology & Metabolism, Ghent University Hospital, 9000 Ghent, Belgium; joel.smet@ugent.be (J.S.); boel.depaepe@ugent.be (B.D.P.); maxime.verschoore@ugent.be (M.V.); 3 Laboratory for Mitochondrial Investigations, Ghent University, 9000 Ghent, Belgium; 4 Department of Pediatric Metabolism, Hacettepe University Ihsan Dogramaci Children’s Hospital, Hacettepe 06230, Turkey; yilmaz.yildiz@hacettepe.edu.tr; 5 Pediatric Metabolic Diseases Unit, Gülhane Training and Research Hospital of the University of Health Sciences, Gülhane 06010, Turkey; 6 Department of Pediatric Neurology, Gülhane Training and Research Hospital of the University of Health Sciences, Gülhane 06010, Turkey; mutluayarslan@yahoo.com; 7 Department of Neurology, Maria Middelares General Hospital, 9000 Ghent, Belgium; olivier.stevens@azmmsj.be; 8 Research Group Reproduction and Genetics (REGE), Free University Brussels (VUB), 1090 Brussels, Belgium; sara.seneca@uzbrussel.be; 9 Centre for Medical Genetics, University Hospital Brussels, Free University Brussels (VUB), 1090 Brussels, Belgium

**Keywords:** ATP-synthase, complex V deficiency, *MT-ATP6*, NC_012920.1(MT-ATP6):m.9035T>C, p.L170P, genotype-phenotype correlation, mitochondrial disorder

## Abstract

Human mitochondrial disease exhibits large variation of clinical phenotypes, even in patients with the same causative gene defect. We illustrate this heterogeneity by confronting clinical and biochemical data of two patients with the uncommon pathogenic homoplasmic NC_012920.1(MT-ATP6):m.9035T>C variant in *MT-ATP6*. Patient 1 presented as a toddler with severe motor and speech delay and spastic ataxia without extra-neurologic involvement. Patient 2 presented in adolescence with ataxia and ophthalmoplegia without cognitive or motor impairment. Respiratory chain complex activities were normal in cultured skin fibroblasts from both patients when calculated as ratios over citrate synthase activity. Native gels found presence of subcomplexes of complex V in fibroblast and/or skeletal muscle. Bioenergetic measurements in fibroblasts from both patients detected reduced spare respiratory capacities and altered extracellular acidification rates, revealing a switch from mitochondrial respiration to glycolysis to uphold ATP production. Thus, in contrast to the differing disease presentation, biochemical evidence of mitochondrial deficiency turned out quite similar. We conclude that biochemical analysis remains a valuable tool to confirm the genetic diagnosis of mitochondrial disease, especially in patients with new gene variants or atypical clinical presentation.

## 1. Introduction

Adenosine triphosphate (ATP) is the main energy source for metabolic processes and is predominantly produced by oxidative phosphorylation (OXPHOS). The OXPHOS system, which is located in the inner mitochondrial membrane, consists of five multiprotein complexes. Complexes I-IV transport protons into the mitochondrial intermembrane space, thereby creating an electrochemical membrane potential. The latter is the driving force allowing complex V (or ATP synthase) to generate ATP from adenosine diphosphate (ADP) and inorganic phosphate. Complex V consists of 17 subunits that compose two functional domains: a membrane embedded part (F_0_) that is connected to the matrix located catalytical domain (F_1_), where ATP is actually produced. Both domains are connected via two stalks that couple proton transfer in F_0_ to ATP synthesis in F_1_ [1].

OXPHOS defects result in mitochondrial disorders. Of these, complex V deficiencies are considered very rare [1]. The most common gene associated with complex V deficiencies, is *MT-ATP6* (OMIM *516060), which encodes subunit a of the F_0_ functional domain [1,2]. This subunit contains the proton pore that releases the proton gradient by conducting protons from the intermembrane space to the matrix. Due to the close interaction between subunit a and the nearby ring of c-subunits, this process leads to the rotation of the latter. The resulting mechanical energy is used to induce conformational changes in the F_1_ domain that favor ATP production [1]. Furthermore, subunit a has also been suggested to play a role in the stabilization and dimerization of complex V [3,4].

Pathogenic variants in *MT-ATP6* are a well-known cause of maternally-inherited mitochondrial disorders [5,6]. However, in a minority of cases de novo mutations have been described as well [7]. The pathogenic variant NC_012920.1(MT-ATP6):m.8993T>G in which a thymine to guanine transversion occurs at nucleotide position 8993, located at the ATP6 locus on the mitochondrial DNA (mtDNA), was one of the first described mtDNA variants and is the most prevalent variant in patients with ATP6 deficiencies [5,6]. Furthermore, 18 additional pathogenic *MT-ATP6* variants have been reported so far, of which several have only been described in a single pedigree [5]. Several variants of unknown significance (VUS) have been reported in the *MT-ATP6* gene as well.

Extensive phenotypic heterogeneity exists among distinct *MT-ATP6* variants and in the accompanying biochemical features [1,5,8,9,10]. The phenotypic spectrum ranges from asymptomatic carriers to fatal early onset and multi-systemic diseases. *MT-ATP6*–related mitochondrial disease has been associated with various phenotypes, including NARP (neuropathy, ataxia, and retinitis pigmentosa) syndrome, Leigh syndrome (LS), Charcot–Marie–Tooth (CMT) disease, spinocerebellar ataxia (SCA), encephalopathy, peripheral neuropathy, proximal myopathy, cardiomyopathy, dystonia. dysarthria, ptosis, external ophthalmoplegia, retinitis pigmentosa (RP), sensorineural deafness, developmental delay, learning disability and exercise intolerance.

Interestingly, even in patients with the same causative gene defect, human mitochondrial disease exhibits a large variation of clinical phenotypes [11]. We illustrate this heterogeneity by comparing the clinical and biochemical data of two patients with the uncommon pathogenic homoplasmic variant NC_012920.1(MT-ATP6):m.9035T>C. This variant converts leucine-170 to proline in subunit a. Its secondary structure predicts that this amino acid is located within a transmembrane helix of subunit a, more specifically, at the critical interface of c subunit contact [9]. This variant was first described in 2009 by Sikorska et al. in patients with cognitive delay, and early-onset ataxia [12]. Its pathogenicity was demonstrated using transmitochondrial cybrids. Furthermore, it has also been associated with adult-onset ataxia [13]. Overall, only a few papers have reported on patients with NC_012920.1(MT-ATP6):m.9035T>C [5,8,12,13,14,15,16,17] (see Table S1 for an overview of previously published cases).

## 2. Materials and Methods

### 2.1. Patient Description

#### 2.1.1. Clinical Presentation and Physical Examination

Patient 1 (P1) reportedly displayed the first signs of disease at six months of age, when his parents noticed that he was unable to sit upright and appeared to have significant stiffness of all limbs, particularly of the lower extremities. Eventually, the boy was able to sit without support by the age of 10 months and learned to stand briefly at an age of 18 months. At presentation to the metabolic clinic, at the age of two years, the patient demonstrated severe motor and speech delay, and spastic ataxia without extra-neurological involvement. The child could not walk independently and supported himself with a wide stance. Occasionally, he used two-word sentences, but with poor articulation. Physical examination confirmed hypertonicity in all, but predominantly the lower, extremities as well as the presence of truncal hypotonia. Further investigations showed a normal head circumference (48 cm; z-score: −0.47), hyperactive deep tendon reflexes (no ankle clonus), bilateral extensor plantar responses, and a spastic scissoring posture of the lower limbs during vertical suspension. Anamnesis revealed that the patient had a maternal half-sister, who was born at 32 weeks of gestation due to preterm labor with a birth weight of 2000 g (z-score: 0.45), and also had a severe language and cognitive delay. She developed epilepsy three months after birth, could walk independently at 18 months, and suffered from recurrent pulmonary infections and vomiting. She succumbed to pneumonia at the age of 22 months.

Patient 2 (P2) first presented to the emergency department at 19 years of age. She reported progressive visual impairment over the last couple of weeks, as well as the occurrence of diplopia. Upon examination both pupils were mydriatic and displayed minimal light reactivity. Bilateral internuclear ophthalmoplegia was observed, as well as a conjugate upward gaze palsy with bilateral ptosis. The patient appeared well oriented, did not display any phatic disorders nor cognitive impairment. No facial numbness or paralysis of the limbs could be observed. Furthermore, the patient had an atactic gait pattern, a decreased sense of balance and could not walk without support. The heel-to-shin test revealed light appendicular ataxia. Tendon reflexes were brisk, but did not appear to be pathological. Bilateral extensor plantar responses could be observed. In addition, the patient displayed urinary retention for which she required an indwelling catheter. The patient’s previous medical history was unremarkable. Interestingly, both the patient’s mother, one of two maternal uncles and her maternal grandmother reported balance disorders and gait abnormalities as well. After several weeks the patient’s ability to walk, as well as the gaze palsy, gradually improved, although a multidirectional nystagmus remained.

#### 2.1.2. Technical Investigations

**Figure 1:**
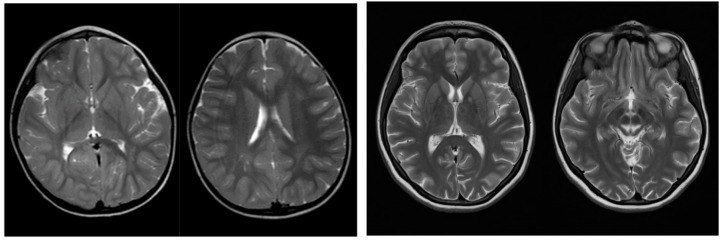
Axial T2-weighted magnetic resonance images of P1 (**left panel**) and P2 (**right panel**), respectively. For P1, zones of late myelination were observed (second image of the **left panel**), whereas for P2 T2 hyperintensities resembling Panda sign could be discerned (second image of **right panel**).

T2-weighted and fluid-attenuated inversion recovery (FLAIR) brain magnetic resonance (MR) imaging of P1, performed at the age of three years, showed small, bilaterally symmetric hyperintensities in the parietal white matter, most compatible with zones of late myelination (see Figure 1, left panel). Furthermore, MR-spectroscopy did not show any abnormalities in biochemical tissue composition.

The cerebral MR imaging of P2 showed pronounced, symmetrical T2/FLAIR hyperintensities of the anterior part of the globus pallidus, peri-aqueductal grey matter, the cerebellum, the posterior part of the pons and the reticular formation of the mesencephalon, resembling a Panda sign (see Figure 1, right panel). This sign may be suggestive of Wilson’s disease or other causes of diffusely abnormal white matter. Furthermore, MR spectroscopy detected an increased lactate concentration in the brain stem, potentially suggesting the presence of an underlying metabolic disorder. Further follow-up MR imaging after several weeks and months confirmed these findings, although a gradual decrease of the abovementioned abnormalities could be observed. MR imaging of the spinal cord performed during the initial presentation, did not show any abnormalities. The visual evoked potential (VEP) test was normal, suggesting correctly functioning nerve pathways of the optic system. Further ophthalmologic evaluation including fundoscopy and fluorescein angiography did not reveal any specific abnormalities. A disturbed eye motility was the only aberrancy that could be observed during these investigations. In particular, no Kayser Fleischer rings could be discerned. In addition, sonographic evaluation of the liver could not reveal any abnormalities.

#### 2.1.3. Laboratory Investigations

To investigate the presence of a potential underlying metabolic disorder, an extensive metabolic screening was performed for P1. This included the determination of blood gases, plasma lactate and plasma ammonia concentrations, the assessment of the plasma amino acid and urinary amino acid and organic acid profiles, as well as acylcarnitine profile analysis on dried blood spots, quantitation of very long chain fatty acids and phytanic acid in plasma, and evaluation of lysosomal enzyme activities (including arylsulfatase A, β-galactocerebrosidase and β-galactosidase) in leukocytes. Furthermore, transferrin isoelectric focusing was performed on serum. None of these analyses revealed any significant abnormalities.

The biochemical work up of P2 showed a normal serum creatine kinase activity, a normal plasma lactate concentration and moderately increased plasma ammonia (73 µmol/L, ref: 11–48), transaminases (ALT: 121 IU/L, ref: 7–3; AST: 59 IU/L, ref: 0–31) and erythrocyte sedimentation rate (51 mm/h, ref: 0–20). Aside from a slightly increased urinary lactate concentration (212 mmol/mol creatinine, ref: < 61), no abnormalities could be discerned in the organic acid profile. The plasma amino acid and acylcarnitine profiles did not show any significant abnormalities. Furthermore, the white blood cell count, the total protein and lactate concentration determined on cerebrospinal fluid were normal. Corresponding viral PCRs and bacteriological cultures were all negative. Auto-immunity screening including anti-ganglioside antibodies, (paraneoplastic) anti-neuronal antibodies, extractable nuclear antigen antibodies (ENA) and anti-neutrophil cytoplasmic antibodies (ANCA), proved to be negative. To assess the possibility of Wilson’s disease the copper metabolism was evaluated. The serum copper concentration was normal and the ceruloplasmin level was slightly decreased. The 24 h copper excretion in urine was very low at baseline (i.e., 20 µg/24 h) and the post-penicillamine urinary copper excretion was 883 µg/24 h, findings not suggestive of Wilson’s disease. In addition, genetic testing for Wilson’s disease, performed via next-generation sequencing of all coding exons of the ATP7B gene (OMIM *606882), could not withhold any pathological variants. In addition, whole blood vitamin B1 concentration was normal, further excluding Wernicke encephalopathy.

### 2.2. Biopsies

To evaluate the possibility of a mitochondrial disorder, skin biopsies were performed in both patients. From the latter, a fibroblast cell culture was derived. Fibroblasts were cultured using Opti-MEM medium supplemented with 20% fetal bovine serum, 0.5 U/mL penicillin-streptomycin and 1% kanamycin (ThermoFisher Scientific, Waltham, MA, USA) in a humidified incubator with 5% CO_2_ at 37 °C. For P2, a skeletal muscle biopsy was performed as well. Tissue specific control material for all biochemical analyses was obtained from both adult and pediatric patients without proven evidence of mitochondrial or other neurological disease. This control material was used to set up reference intervals and/or as internal quality control. The latter were not age-matched. Ethical approval was obtained from the Ghent University Institutional Review Board, and all patients or patient’s guardians signed an informed consent.

### 2.3. Next-Generation Sequencing of the Mitochondrial Genome

Next-generation sequencing (NGS) of the mitochondrial genome was performed on blood leucocytes and skin fibroblasts of both patients, as well as on skeletal muscle of P2. Blood leucocytes from the mother of P1 were also analyzed. Total DNA was extracted from leukocytes using standard DNA isolation techniques (Chemagen, Perkin Elmer, Zaventem, Belgium). DNA from fibroblast culture and muscle tissue was isolated by a laboratory developed protocol of proteinase K–SDS lysis, followed by phenol–chloroform extraction and ethanol precipitation [18].

The mitochondrial genome of a DNA sample (200 ng) was amplified with Long Range PCR methodology as one very large fragment following the manufactur’s instructions of the LongAmp Taq DNA polymerase kit (Bioke, Leiden, Nederland). After electrophoresis and data analysis of the long range PCR amplicon size on a Fragment Analyzer device (Advanced Analytical, Fiorenzuola d’Arda, Italy), the large amplicon was fragmented to an average length of around 220 bp by mechanical shearing with a Covaris M220 sonicator (Covaris Life Technologies Europe, Bleiswijk, Nederland). Sheared samples were purified using AMPure beads. Subsequently, a DNA library was prepared according to the manufacturer’s instructions of the KAPA Hyper preparation kit (KAPA Biosystems, Woburn, MA, USA). After fragmentation, end repair, adenylation, and indexed paired-end adapter ligation, samples were pooled and processed for sequencing. The fragments were amplified and sequenced in 2 times 100 bp paired-end modus on a NovaSeq 6000 machine (Illumina, San Diego, CA, USA). Data processing (annotation, variant detection and coverage) of the sequenced regions was performed with a laboratory developed pipeline. The coverage of all regions analyzed has to be, at least, 5000 reads but it largely exceeded this treshold. A general detection limit of > 3% was used. Sequencing data was classified according to the 5 class (CL) system: benign (CL1), likely benign (CL2), variant of unknown significance (CL3 or VUS), likely pathogenic (CL4) and pathogenic (CL5). This classification was performed according to information published in databases (www.mitomap.org, accessed on 30 October 2019) and reported in the literature. Class 4 and 5 variants were confirmed with Sanger sequencing (always with the exception of (very) low heteroplasmic variant loads, which are under the Sanger sequencing detection limit). All nomenclature was according to the rCRS sequence with number NC_012920.1 and conform HGVS guidelines (www.hgvs.org, accessed on 30 October 2019).

### 2.4. Evaluation of Mitochondrial Function

#### 2.4.1. Spectrometry

The catalytic activities of citrate synthase and of the OXPHOS complexes I, II, II+III, III and IV in skeletal muscle homogenate and mitochondria isolated from skeletal muscle, as well as the activities of the OXPHOS complexes II, II+III, III and IV in fibroblast homogenates, were measured by spectrophotometric analysis, as previously described [19,20,21,22,23,24,25,26].

The oligomycin sensitive fraction of complex V was determined on isolated mitochondria from skeletal muscle and cultured skin fibroblasts. The utilized method was adapted from previously published procedures [22,27]. Briefly, the isolated mitochondria were incubated in a mixture of 40 mM Tris buffer (pH 8.0), 10 mM potassium chloride (KCl), 5 mM magnesium chloride (MgCl_2_), 0.1% bovine serum albumin (BSA), 2 mM phosphoenol pyruvate, 0.2 mM nicotinamide adenine dinucleotide (NADH), 6 U/mL pyruvate kinase/lactate dehydrogenase, 1 nM antimycin, and 3 nM carbonyl cyanide 3-chlorophenyl hydrazine (CCCP) at 37 °C for 2 min. Subsequently, and in contrast to the previously published procedures mentioned above, ATP was added in a separate step to a concentration of 0.5 mM. From that moment on, the decrease in absorbance at 340 nm was monitored to determine the rate of NADH oxidation, equimolar to ATP hydrolysis. After 4 min, 3 nM oligomycin, an inhibitor of complex V, was added and the absorbance at 340 nm was measured to determine the oligomycin insensitive ATPase activity. The difference between the total ATPase activity and the oligomycin insensitive fraction, respectively, yields the oligomycin sensitive ATPase activity.

Activities of the OXPHOS complexes are expressed as nanomoles per minute per mg of protein. These are further normalized to the activity of citrate synthase to control for mitochondrial mass. These activity ratios are obtained by calculating the ratio of the logarithm of complex activity over the logarithm of citrate synthase activity. Subsequently, the z-score of each complex is calculated as the activity ratio for the patient sample minus the mean activity ratio of the control samples divided by the standard deviation (SD) of the activity ratios of the control samples. If the z-score is lower than −1.96 or higher than +1.96, the patient sample is significantly different (*p* < 0.05) from the control samples.

#### 2.4.2. Respirometry

Oxygen consumption rates (OCR, expressed as pmol/min) and extracellular acidification rates (ECAR, expressed as mpH/min) of cultured skin fibroblasts were determined using a Seahorse XFp device (Agilent, Santa Clara, CA, USA) and Seahorse CF Cell Mito Stress Test kit. For each patient or control sample three wells were seeded with 30,000 cells each and cultured for 24 h in Opti-MEM medium (ThermoFisher Scientific). Subsequently, the medium was replaced with standard Seahorse assay medium supplemented with 10 mM glucose, 1 mM pyruvate and 2 mM glutamine (Sigma, St. Louis, MO, USA). Cells were consecutively challenged with 4 µM oligomycin, 2 µM carbonyl cyanide 4-(trifluoromethoxy)-phenylhydrazone (FCCP) and 0.5 µM of a rotenone antimycin A mix (Agilent). At baseline and after each challenge, ECAR and OCR were measured in triplicate. The different OCR and ECAR derived bioenergetic parameters were calculated as previously described [28]. Briefly, the basal respiration was calculated as the OCR before addition of stressors/inhibitors minus the non-mitochondrial respiration rate. The latter was determined after challenge with the rotenone antimycin A mix. The maximal respiration was calculated as the OCR after FCCP challenge, minus the non-mitochondrial respiration rate, and the spare respiratory capacity was calculated as the difference between the basal and the maximal (FCCP-provoked) OCR. The ATP-linked respiration was calculated as the difference between the OCR before addition of stressors/inhibitors and the OCR after addition of oligomycin. Baseline OCR/ECAR and stressed OCR/ECAR ratios are determined before addition of stressors/inhibitors, and after FCCP challenge, respectively. The metabolic potential was calculated as the ratio of the stressed ECAR over the baseline ECAR. The basal respiration, maximal respiration, spare respiratory capacity and ATP production were all evaluated as such, after normalization against total protein content (µg/well) and CS activity (nmol/min), respectively. This strategy is based on a previously published paper by Panneman et al. [29]. To that end, fibroblasts were detached from the wells by trypsinization, washed with isotonic saline solution, pelleted through centrifugation at 16100 g for 15 min and re-solubilized in 100 µL of isotonic saline solution. Ten microliter of this solution was used to determine the protein amount using the Pierce^TM^ Coomassie (Bradford) Protein assay kit (Catalogue 23200, ThermoFisher Scientific) [30]. Eighty microliter was used for citrate synthase measurement according to the method described by Srere et al. [25]. The bioenergetic evaluation was performed in duplicate for both patients.

#### 2.4.3. Blue Native PAGE with in Gel Activity Staining

**Figure 2:**
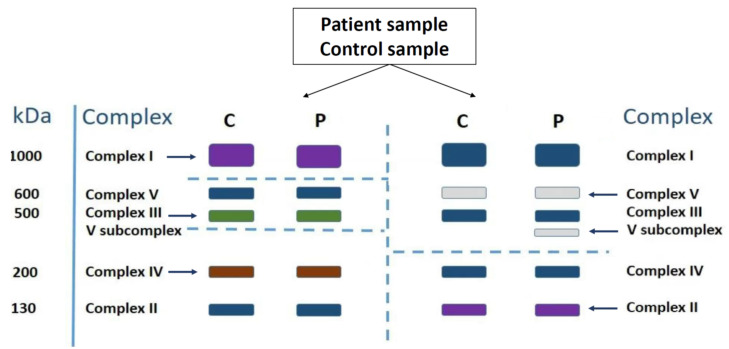
Schematic representation of BN-PAGE with in-gel activity staining. Dashed lines represent how the gel is cut into five fragments. The arrows indicate which OXPHOS complex activity is visualized in which gel fragment. P = patient sample, C = control sample.

Mitochondria were isolated from skeletal muscle tissue and from cultured skin fibroblasts. For fibroblasts approximately 50 µg of mitochondrial protein was loaded, whereas for skeletal muscle approximately 60 µg of mitochondrial protein was loaded. An equal amount of mitochondrial protein was loaded of both patient and control sample. The OXPHOS complexes were separated by Blue Native-Polyacrylamide Gel Electrophoresis (BN-PAGE) followed by in-gel catalytic activity staining, performed according to previously published methods [31,32]. Briefly, after separation, two lanes per patient and tissue-specific control sample were used for catalytic in-gel activity staining. Due to the coupling to Coomassie blue G, the OXPHOS complexes were visualized. This served as a guidance, to subdivide the gel in five different fragments. The latter is required, as the catalytic activity staining of the different OXPHOS complexes requires different incubation procedures. The BN PAGE gel is first cut vertically and the left fragment is further cut between complex I and V and underneath complex III, yielding three gel fragments. The upper part is used for complex I staining, the middle part for complex III staining, and the lower part for complex IV staining. The right fragment is cut between complex III and IV. The upper part is used to stain complex V and, if present, complex V subcomplexes. The lower part serves for complex II staining. A schematic representation of this process can be seen in Figure 2.

#### 2.4.4. Two-Dimensional Gel Electrophoresis and Western Blotting

Mitochondrial protein fractions were isolated from skeletal muscle and cultured skin fibroblasts, and separated in the first dimension using BN-PAGE. Subsequently, tricine sodium dodecyl sulphate polyacrylamide gel electrophoresis (t-SDS-PAGE) was performed to separate the protein subunits within each OXPHOS complex in a second dimension, perpendicular to the first one [33,34]. Proteins were transferred by electroblotting onto a nitrocellulose membrane (Amersham Biosciences, Uppsala, Sweden). Non-specific antibody binding was blocked using 5% nonfat dry milk (Bio-Rad, Temse, Belgium) in phosphate-buffered saline with 0.1% Tween 20. Immunoblotting of the OXPHOS complexes was carried out with the total OXPHOS human WB antibody cocktail ab110411 (Abcam, Cambridge, UK), which contains five mouse monoclonal antibodies, one against a subunit of each OXPHOS complex. More specifically, these antibodies target NADH:ubiquinone oxidoreductase subunit B8 (I-NDUFB8), succinate dehydrogenase subunit B (II-SDHB, also called the iron-sulfur protein subunit or IP), ubiquinol-cytochrome C reductase core protein 2 (III-UQCRC2), cytochrome c oxidase subunit 2 (IV-COX II), and ATP synthase subunit alpha (V-ATP5A). Detection was achieved using a secondary anti-mouse IgG coupled to horse radish peroxidase (GE Healthcare, Machelen, Belgium) in combination with the chemiluminescence Pierce ECL Plus substrate (ThermoFisher Scientific) according to the manufacturer’s instructions, visualized using a Celvin analyzer, and analyzed with SnapAndGo software (Biostep, Burkhardtsdorf, Germany).

After visualisation of the respective subunits, the blot was stripped by incubating the membranes for 30 min. in a solution containing 2 m/v% sodiumdodecylsulphate (SDS) and 0.05 M dithiotreitol (DTT) in a 0.05 M Tris buffer at 50 °C and reincubated with anti-ATP6 (Merck, Overijse, Belgium), to evaluate the amount of ATP6 protein present.

### 2.5. Microscopy

On skeletal muscle sections from P2 standard histological stainings were performed.

Fibroblasts from both patients were cultured on glass chamber slides for fluorescent imaging (Nunc, Rochester, NY) until a confluence range of 60 to 80%. The mitochondrial membrane potential was evaluated using a previously published fluorescence imaging method [35]. Red over green fluorescence ratio of the cationic dye 5,5′, 6,6′-tetraethylbenzimidazolyl-carbocyanine iodide (JC-1) (ThermoFisher Scientific) was visualized with and without preincubation with rotenone. Ten microscopic fields ×400 magnification were visualized to calculate the average JC-1 ratio and corresponding SD.

In addition, cells were immunostained with 0.2 µg/mL rabbit polyclonal anti-ATP6 antibody (SAB5700851, Merck) for 2 h at room temperature, and visualized with an anti-rabbit secondary antibody labeled with AlexaFluor488 (ThermoFisher Scientific). Double staining visualized mitochondria with the membrane potential-dependent fluorescent Mitotracker^TM^ Red CMXRos dye (ThermoFisher Scientific). Slides were mounted with vectashield containing 4′-6-diamidino-2-phenylindole (DAPI) to stain nuclei (Vector, Burlingame, CA, USA). The ATP6 immunostaining signal was quantified by converting the fluorescent signal to bright field and measuring average grey values in ten random microscopic fields, which were normalized to the DAPI signal, representing the number of cells in the microscopic field, using CellF Soft Imaging System (Olympus, Muenster, Germany).

## 3. Results

### 3.1. Next-Generation Sequencing of the Mitochondrial Genome

In P1, next-generation sequencing of mtDNA revealed a homoplasmic NC_012920.1(MT-ATP6):m.9035T>C variant in blood leukocytes and skin fibroblasts. Skeletal muscle biopsy was not available for testing. Additional homoplasmic m.4216C>T and m.4917A>G polymorphisms were also present. Leukocytes of the healthy mother showed 31% heteroplasmy for NC_012920.1(MT-ATP6):m.9035T>C and homoplasmy for the two polymorphisms. No tissue or genetic material from the deceased half-sister was available for testing.

Blood leukocytes, fibroblasts and skeletal muscle of P2 also carried a homoplasmic NC_012920.1(MT-ATP6):m.9035T>C variant. An additional variant of unknown significance NC_012920.1(MT-ND1):m.3337G>A was found and scored a class 3. The affected residue is located on the NADH dehydrogenase 1 (*MTND1)* gene (*516000 OMIM) [36]. NADH dehydrogenase 1 enables electron transfer from NADH to ubiquinone in complex I. The above-mentioned variant causes substitution of a poorly conserved valine amino acid at position 11 by an isoleucine residue. As the activity of complex I was tested normal (cf. infra), the pathogenicity of this variant was considered unlikely.

### 3.2. Spectrophotometry

No deficiency in OXPHOS complex I, II, II+III, III or IV could be observed in cultured skin fibroblasts from both patients, nor in the skeletal muscle and mitochondria isolated from the skeletal muscle of P2 (see Table 1). The oligomycin sensitive ATPase activity was found to be normal in mitochondria isolated from skeletal muscle from P2. In mitochondria isolated from skin fibroblasts of both patients no decrease in oligomycin sensitive ATPase activity could be discerned either (see Table 1).

### 3.3. Respirometry

**Figure 3:**
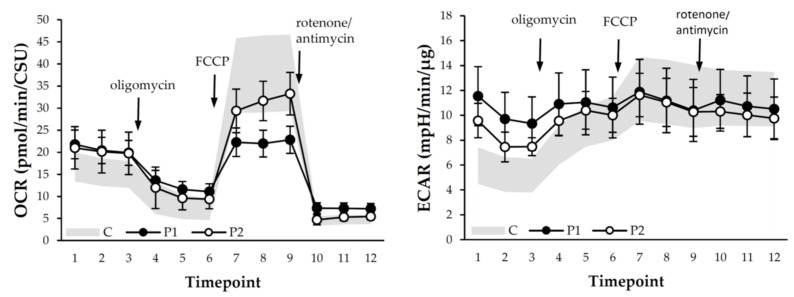
Graphical representation of the oxygen consumption rate (OCR) and extracellular acidification rate (ECAR) at baseline and after challenges with oligomycin, FCCP, and a rotenone antimycin A mix, respectively. OCR is normalized against citrate synthase units (CSU). Depicted are the mean results ± standard deviation (*n* = 5 for P1, *n* = 6 for P2). Filled circles represent the results of P1, unfilled circles the results of P2. The results of the control group (*n* = 20) are depicted in grey.

P1 shows a strongly decreased mitochondrial respiration with a decreased spare respiratory capacity, indicating a decreased ability of the fibroblasts to increase respiration rate in response to energetic demands (see Table 2). Similar results are obtained with and without normalization to total protein content or CS activity. The baseline ECAR is elevated, resulting in a decreased baseline OCR/ECAR ratio. These findings, along with a decreased ECAR metabolic potential is indicative of a pronounced switch to glycolysis as main energy source. As the cells are already heavily dependent on glycolysis at baseline, their glycolytic activity shows only a minimal response to additional stressors. Although similar results were found for P2, these were much less pronounced. The spare respiratory capacity was mildly decreased to low normal (depending on the normalization strategy) and the ECAR metabolic potential and OCR/ECAR ratio were moderately decreased, suggesting a switch to glycolysis for this patient as well. A graphical representation of these results can be seen in Figure 3.

### 3.4. Blue Native PAGE with in Gel Activity Staining

**Figure 4:**
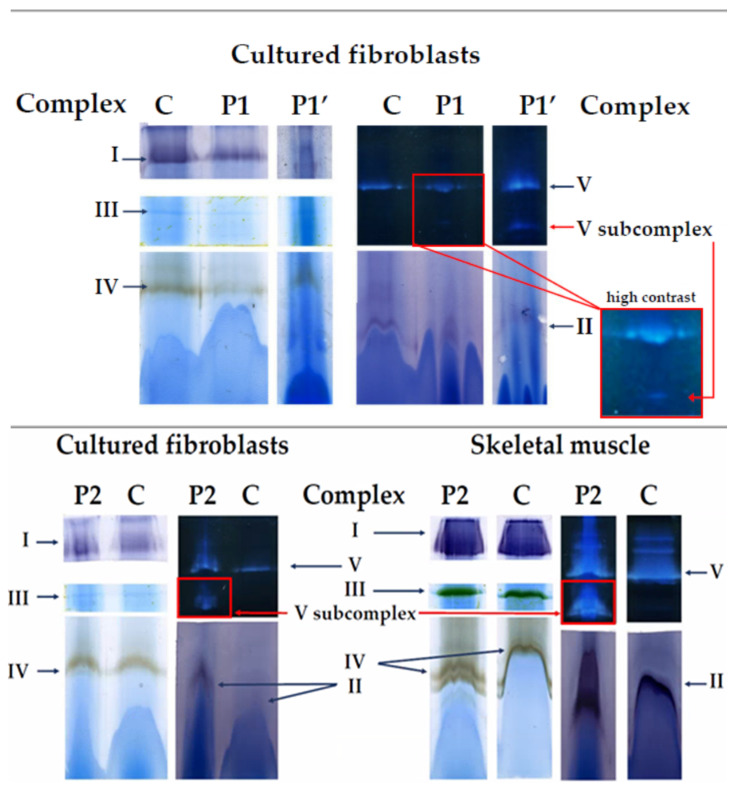
Depicted are the results of BN-PAGE with in gel activity staining. In the **upper panel** the results obtained from mitochondria isolated from fibroblasts of P1 are depicted. These results were confirmed in a separate, second analysis (P1′) starting from more material (approximately 55 vs. 45 µg of mitochondrial protein was loaded. The results for the mitochondria isolated from the fibroblasts and skeletal muscle of P2 are displayed in the **bottom panel**. In each panel, the result of a tissue specific control sample (C), run in parallel, is shown as well. For P2, the amount of mitochondrial protein loaded was approximately 50 µg for fibroblasts and 60 µg for skeletal muscle. Complex V subcomplexes are indicated using red arrows. All other OXPHOS complexes are indicated using blue arrows.

Normal activity staining of the OXPHOS complexes I, II, III and IV was observed for the isolated mitochondria from skeletal muscle and fibroblasts, which confirmed the spectrophotometric results. Catalytically active subcomplexes of complex V could be observed in the fibroblasts of both patients, as well as in the skeletal muscle of P2 (see Figure 4, bottom panel). For P2, presence of a large subcomplex was found, whereas for P1 the presence of the complex V subcomplex could only be discerned when using high contrast imaging (see insert Figure 4, upper panel). Therefore, the latter analysis was repeated starting from more material (indicated as P1’), which showed the presence of a complex V subcomplex more clearly, confirming the previous results.

### 3.5. Two-Dimensional Gel Electrophoresis and Western Blotting

**Figure 5:**
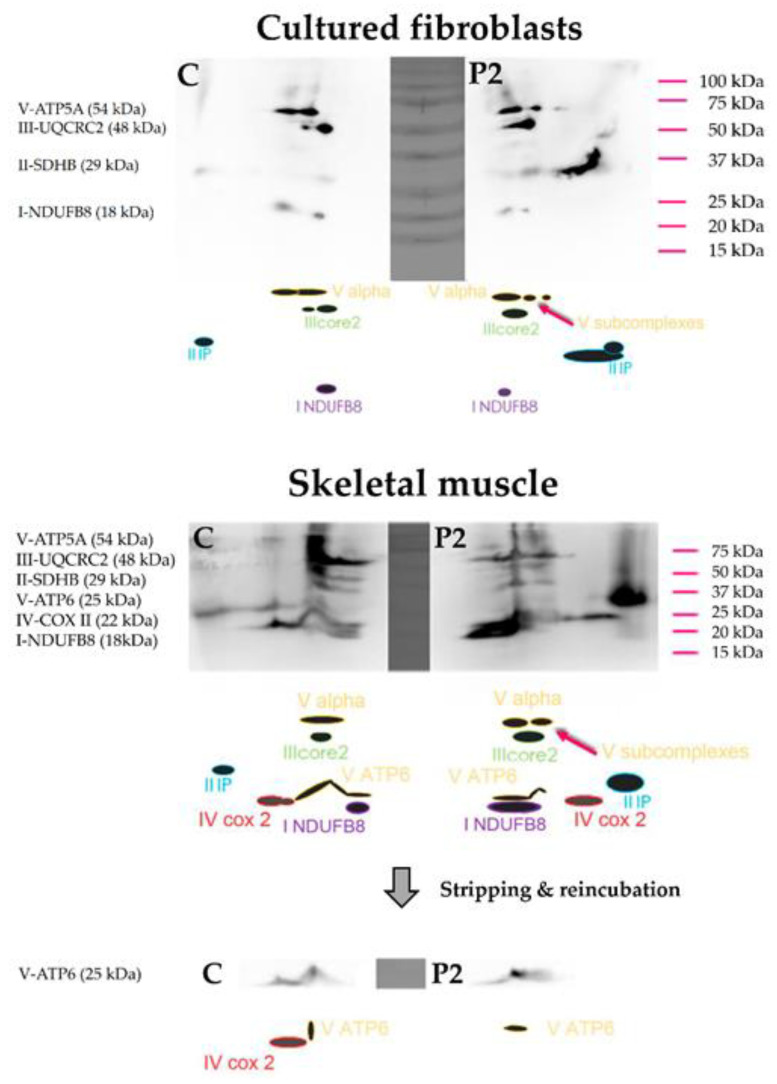
Depicted are the results of the 2D western blot of P2. The results obtained for the isolated mitochondria of cultured skin fibroblasts and skeletal muscle are shown in the **upper** and **middle panel**, respectively. In the **bottom panel** the results after stripping and reincubation with anti-ATP6 are displayed. In each panel the results of a parallelly analyzed tissue specific control sample (C) are included as well.

The two-dimensional (2D) western blots of the cultured skin fibroblasts of P2 demonstrated the presence of complex V subcomplexes, detected using immunoblotting of the alpha subunit of complex V (see Figure 5, upper panel). Unfortunately, due to a disturbance during the 2D electrophoresis, the V-ATP5A and III-UQCRC2 subunits in the fibroblast control sample each appear to have split into two separate protein spots, complicating interpretation of the immunoblot. The presence of complex V subcomplexes was clearly confirmed in skeletal muscle (see Figure 5, middle panel).

For better interpretation of additional ATP6 staining, which has a similar molecular weight as the complex IV-COX II subunit, antibodies were stripped and the blot was re-incubated with anti-ATP6 antibody (see Figure 5, bottom panel). An ATP6 protein spot could be observed at the expected location, both in patient and control (with the control displaying some interference of incompletely stripped anti IV-COX II). The obtained results suggest that the ATP6 expression in the skeletal muscle of P2 is not markedly decreased compared to the control sample. Unfortunately, this analysis could not be performed for the fibroblasts of P1, due to the limited amount of available material.

### 3.6. Microscopic Evaluation

**Figure 6:**
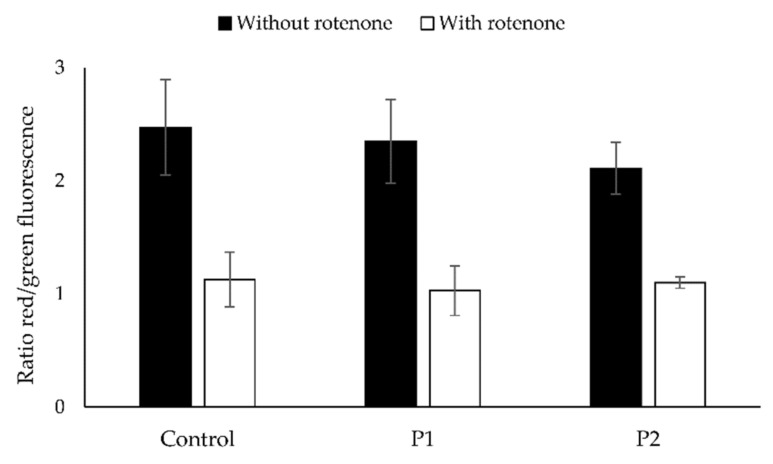
Ratios of red/green fluorescence of both the control sample and the patient samples without and with preincubation with rotenone. Depicted is the average JC-1 ratio ± SD (*n* = 10).

**Figure 7:**
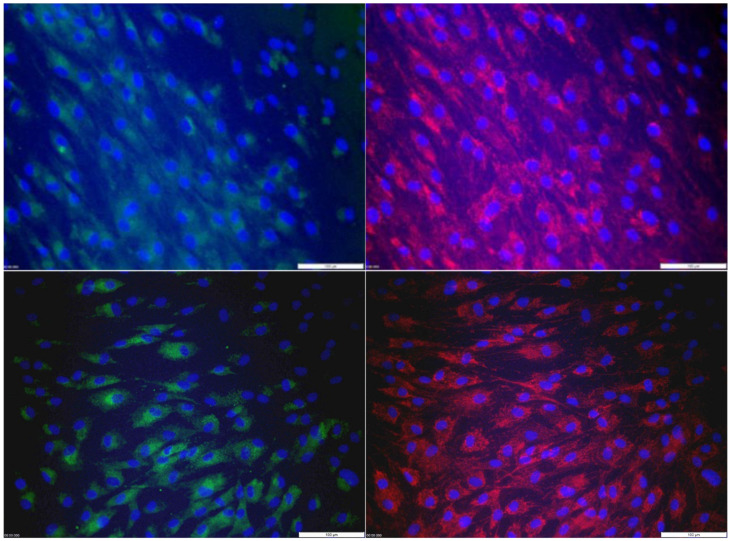
ATP6 immunostaining (**left panels**) and Mitotracker^TM^ staining (**right panels**) counterstained with DAPI of P2 (two **upper panels**) and a control sample (two **bottom panels**).

No abnormalities could be observed upon microscopic evaluation of the muscle sections of P2. Fluorescent cytostaining of cultured skin fibroblasts from patient P2 revealed normal JC-1 ratios and staining patterns (Figure 6). No statistically significant difference could be discerned between the JC-1 ratios of patients and control (α = 0.05). Mitotracker^TM^ staining intensity and ATP6 immunostaining pattern were normal (Figure 7). The ATP6 staining intensity in P2 (1.114 ± 0.139) and the control sample (1.217 ± 0.236) were not statistically different (α = 0.05).

## 4. Discussion

MT-ATP6 related mitochondrial disorders are associated with a wide spectrum of clinical phenotypes [5,8]. Even patients with the same causative gene defect have been known to display large phenotypic heterogeneity [11]. In this paper, we present two patients with the same uncommon, pathogenic NC_012920.1(MT-ATP6):m.9035T>C variant with pronounced differences in clinical presentation. Whereas P1 presented as a toddler with developmental delay and ataxia, adult onset ophthalmoplegia and ataxia characterized the clinical picture of P2. Additionally MR spectroscopy was aberrant for P2, but not for P1.

Mitochondrial disease phenotype severity has often been linked to the degree of heteroplasmy, although this correlation is all but absolute [5,6,11,14,37]. For NC_012920.1(MT-ATP6):m.9035T>C, for example, this correlation does not hold true [15]. Indeed, in the literature, both patients with a more severe presentation during childhood consisting of cognitive developmental delay and progressive ataxia, and patients with a milder phenotype characterized by an adult-onset spinocerebellar syndrome, displayed very high levels of heteroplasmy (90–100%) [12,13,14,15,16,17]. In our study as well, despite the different clinical presentation, the NC_012920.1(MT-ATP6):m.9035T>C pathogenic variant was found to be homoplasmic in the evaluated tissues of both patients. Furthermore, the NC_012920.1(MT-ATP6):m.9035T>C variant was also found in blood leukocytes from the asymptomatic mother of P1, at a heteroplasmy level of 31%. The above mentioned findings suggest there is a very high heteroplasmy threshold required for NC_012920.1(MT-ATP6):m.9035T>C to trigger phenotypic manifestations, but once this threshold is exceeded, this can result in different clinical manifestations. On average, though, a more severe clinical presentation has been associated with a lower age of onset, which is in line with the observations in our patients as well [8,12,13,14].

This complex genotype/phenotype correlation has been attributed to yet to be determined modifying factors that modulate the biochemical defects and thereby the clinical phenotype [5,38]. Moreover, penetrance of *MT-ATP6* variants, even when homoplasmic, may not be complete [5]. In this study, other mtDNA variants of unknown significance were identified in both patients, but there are no arguments to assume these affect the clinical phenotype. Nonetheless, the different clinical presentation suggests that some additional factors may be present that modulate the phenotypic expression/severity of the NC_012920.1(MT-ATP6):m.9035T>C variant. These factors may be the mtDNA haplogroup, nuclear DNA background, mtDNA copy number or environmental factors, as has been suggested for other mtDNA variants [5,37,38,39,40,41]. Unfortunately, as nuclear DNA analysis was not performed, the influence of potential nuclear DNA variants could not be assessed. Mitochondrial DNA haplogroup and copy number were not determined either. As the mtDNA variant found in both patients is known to increase reactive oxygen species, differences in e.g., cellular compensatory scavenging mechanisms could potentially affect phenotype severity. Furthermore, it needs to be taken into account that the principally affected tissue in these patients (i.e., neuronal cells) cannot be tested, and muscle and cultured skin fibroblasts were used as a surrogate. It cannot be ruled out that differences exist between the different subjects that are only observed in the target tissue and that may correlate with disease severity.

To evaluate the effects of the NC_012920.1(MT-ATP6):m.9035T>C pathogenic variant on both patients on a cellular level, biochemical analyses were performed. Despite the differences in clinical presentation, biochemical analysis turned out rather similar.

As could be expected for an OXPHOS complex V defect, fluorescent evaluation of the mitochondrial membrane potential did not show any abnormalities and respiratory chain complex activities (I, II, II+III, III and IV) were normal when calculated as ratios over citrate synthase activity. Interestingly, OXPHOS activities in cultured skin fibroblasts of both patients were low normal, with values normalizing when calculated as ratios over citrate synthase activity, pointing to a low abundance of mitochondria.

Whereas some have described a decreased oligomycin sensitive ATPase activity in NC_012920.1(MT-ATP6):m.9035T>C cybrids [12], or have suggested that the degree of complex V activity reduction could even be a measure of disease severity [17], no such decrease could be observed in the isolated mitochondria from cultured skin fibroblasts of both patients. Although some have stated muscle to be more sensitive than fibroblasts to the effect on ATPase in case of this variant, evaluation of the ATPase activity in isolated mitochondria of the skeletal muscle of P2 turned out normal as well [15]. It should be stated, however, that for P1, who displayed a more severe clinical phenotype, muscle biopsy was not performed. Despite the spectrophotometric evaluation of ATPase activity being a complex V specific assay, also others have reported it to be a relatively insensitive parameter for complex V defects [5,15]. This is most likely due to the fact that the spectrophotometric assay (which is most commonly used) evaluates the reverse reaction of ATPase, i.e., ATP hydrolysis. The latter reaction does not require proton pumping and therefore does not provide a representative evaluation of the complex V function [5]. In addition, differences in methodology have been known to lead to different results [15]. Although the studies referenced above also evaluated ATPase activity measured as ATP hydrolysis, they did not use isolated mitochondria but permeabilized cybrids or cells. Furthermore, in our study, analysis was performed on frozen tissues, which has been suggested to be less reliable than fresh tissue [42].

Bioenergetic measurements in fibroblasts from both patients detected reduced spare respiratory capacities and altered extracellular acidification rates, revealing a switch from mitochondrial respiration to glycolysis to uphold ATP production. This confirms the findings in previous reports that increased glycolysis is a sensitive (yet non-specific) parameter of deficient aerobic ATP production, thus including complex V defects [15]. Interestingly, the more subtle oximetric alterations for P2, may potentially be a translation of the milder clinical phenotype and the adult onset. Nonetheless, we would like to emphasize that the relationship between these observations is hypothetical and warrants further investigation on a larger patient cohort. It should be specified that although the OCR is typically normalized to total protein, we opted to use citrate synthase as a normalization parameter as well (by analogy with the normalization strategy used for OXPHOS complex activities). Indeed, by normalizing against citrate synthase, a correction is performed for mitochondrial content. The latter is important, as a compensatory increase in mitochondrial content can occur in case of OXPHOS defects, which could lead to seemingly normal bioenergetic results if mitochondrial content is not taken into account. Total protein normalization on the other hand, seems to be the preferred normalization strategy in e.g., mtDNA depletion syndromes, as then the OCR per cell is most informative. Therefore, we opt to use both normalization strategies for OCR data. ECAR results are not normalized against citrate synthase, as glycolysis does not occur in the mitochondria.

Native gels found the presence of subcomplexes of complex V in fibroblasts and/or skeletal muscle of both patients. These findings were confirmed for P2 using two-dimensional electrophoresis and western blotting. Although P1 demonstrated the most severe symptoms, the presence of complex V subcomplexes was more pronounced for P2. Our experience shows, however, that there is no strict correlation between the proportion of subcomplex to holocomplex and the severity of clinical presentation.

As some pathogenic variants of *MT-ATP6* have been known to prevent synthesis of complex V subunit a, or to result in the formation of incomplete ATPase complexes that are capable of ATP hydrolysis but not ATP synthesis, ATP6 expression was evaluated in both fibroblasts and skeletal muscle [43]. Although previous reports observed a decreased ATP6 expression in muscles with NC_012920.1(MT-ATP6):m.9035T>C variant, a marked decrease in ATP6 expression could not be observed in skeletal muscle from P2, using both immunoblotting and immunocytochemical staining [15]. Furthermore, the presence of a substantial amount of complex V holocomplex, detected using BN-page and in gel activity staining, also pleads against a marked decrease in ATP6 expression. However, it should be noted that the employed methodologies are not able to detect more subtle variations in protein expression. These findings seem to favor a functional defect of the catalytic activity of complex V over a defect caused by absent ATP6 due to early protein degradation. The occurrence of subcomplexes suggest that variant NC_012920.1(MT-ATP6):m.9035T>C could cause problems with complex V assembly or stability. However, further research is warranted to further evaluate these hypotheses.

Although next-generation sequencing has become an essential tool in the diagnosis of mitochondrial disorders, functional evaluation remains an important tool in trying to elucidate different clinical presentations, and to evaluate the clinical relevance of new variants of unknown significance. Particularly in the case of *MT-ATP6* variants, in which microscopic analysis and conventional OXPHOS activity (complex I-IV) tend to be unremarkable, and the evaluation of ATPase activity is likely to appear normal as well, validating the pathogenicity of rare or novel variants poses a diagnostic challenge. Especially when these variants are homoplasmic, the distinction between a polymorphism and a pathogenic variant can prove difficult. Here, immunoblotting can be of added value, as it allows to evaluate the presence of subcomplexes and to assess protein abundance. Furthermore, respirometry can provide insight into the effects on aerobic and anaerobic ATP production. Hence, biochemical testing remains a valuable aid to help confirm the genetic diagnose of mitochondrial disease, particularly in patients with new gene variants or atypical clinical presentation.

## 5. Conclusions

The heterogeneity of mitochondrial disease was demonstrated by confronting the clinical and biochemical data of two patients with the uncommon pathogenic homoplasmic NC_012920.1(MT-ATP6):m.9035T>C variant. In contrast to the differing disease presentation, biochemical evidence of mitochondrial deficiency turned out rather similar overall. For both patients, OXPHOS activities (including complex V) were normal, native gels showed the presence of subcomplexes of complex V and bioenergetic measurements showed a switch to glycolysis. Furthermore, ATP6 expression did not appear markedly decreased compared to the control sample, using both immunoblotting and immunocytochemical staining. This could suggest the reported pathogenic variant causes a functional defect of the catalytic activity of complex V rather than a defect caused by absent ATP6 due to early protein degradation. Yet again, biochemical testing has proven to be a valuable asset in confirming the genetic diagnose of mitochondrial disease and in trying to elucidate the underlying mechanism of mitochondrial disorders. This can be of particular added value in patients with new gene variants or atypical clinical presentation.

## Figures and Tables

**Table 1:** OXPHOS complex activities as measured by spectrophotometry and normalized to citrate synthase activity.

Tissue	Patient	Complex I/CS	Complex II/CS	Complex II+III/CS	Complex III/CS	Complex IV/CS	Complex V/CS	Citrate Synthase ^a^
Cultured skin fibroblasts homogenate	P1	ND	0.72 (2.13)	0.80 (2.03)	0.81 (−0.87)	0.97 (0.25)	ND	46
	P2	ND	0.63 (0.43)	0.61 (−0.70)	0.90 (0.44)	0.94 (−0.31)	ND	39
	C (*n* = 30)	ND	0.61 ± 0.05	0.66 ± 0.07	0.87 ± 0.07	0.96 ± 0.05	ND	82 ± 15
Cultured skin fibroblasts isolated mitochondria	P1	ND	ND	ND	ND	ND	0.88 (4.1)	105
	P2	ND	ND	ND	ND	ND	0.81 (2.0)	114
	C (*n* = 4)	ND	ND	ND	ND	ND	0.73± 0.04	242 ± 62
Skeletal muscle homogenate	P2	0.63 (0.13)	0.67 (−0.32)	0.70 (0.39)	0.87 (−0.26)	0.91 (−1.48)	ND	144
	C (*n* = 30)	0.62 ± 0.07	0.68 ± 0.04	0.68 ± 0.04	0.89 ± 0.07	1.00 ± 0.06	ND	174 ± 70
Skeletal muscle isolated mitochondria	P2	0.70 (−0.27)	0.88 (1.17)	0.87 (1.62)	0.96 (1.92)	1.04 (1.66)	0.87 (−0.98)	760
	C (*n* = 30)/ ^b^ (*n* = 20)	0.72 ± 0.06	0.82 ± 0.05	0.79 ± 0.05	0.81 ± 0.08	0.97 ± 0.04	0.95 ± 0.08 ^b^	830 ± 335

^a^ specific activity expressed as nanomoles of substrate per minute per milligram of protein. OXPHOS complex activities are expressed as activity ratios. The corresponding z-score is mentioned between brackets. For the tissue specific control samples (C) the mean activity ± standard deviation is shown, with the amount of control samples indicated between brackets. Results below the corresponding reference interval are indicated in bold. Not determined (ND).

**Table 2:** Results of the bioenergetic measurements.

Parameter	P1	P2	Reference Values				
			Mean	SD	Median	P5	P95
**Normalization Parameters**							
Protein (µg/well)	10.1	7.8	9.0	2.9	8.4	5.9	15.1
CSU (nmol/min)	**373**	**420**	757	228	684	530	1145
**Respiration (OCR)**							
Basal respiration	**47 ± 1**	61 ± 8	77	17	75	58	114
Basal/Protein	**4.8 ± 0.9**	8.0 ± 0.9	9.0	2.2	9.3	5.5	13
Basal/CSU	13 ± 2	15 ± 4	11	2.2	10.0	8.5	14
Maximal respiration	**58 ± 3**	**118 ± 4**	249	37	241	202	309
Maximal/Protein	**5.8 ± 1.0**	**15 ± 3**	29	7.7	29	19	42
Maximal/CSU	**16 ± 2**	29 ± 4	34	7.5	34	23	45
SRC	**11 ± 4**	**56 ± 10**	172	27	171	140	226
SRC/Protein	**1.1 ± 0.3**	**8 ± 2**	20	5.9	21	12	33
SRC/CSU	**2.9 ± 0.9**	14 ± 2	24	5.9	25	14	35
ATP production	**33 ± 1**	43 ± 6	58	13	54	45	70
ATP/Protein	**3.3 ± 0.6**	5.5 ± 0.6	6.7	1.5	6.5	4.7	9.0
ATP/CSU	8.8 ± 1.1	10.5 ± 2.6	7.9	1.6	7.7	5.3	10.7
**Energy Phenotype (ECAR & OCR/ECAR)**							
Baseline ECAR/protein	**9.3 ± 2.2**	**7.5 ± 0.7**	5.1	1.4	4.8	3.4	7.0
ECAR potential	**1.3 ± 0.1**	**1.6 ± 0.1**	2.5	0.3	2.5	1.9	2.9
Baseline OCR/ECAR	**0.8 ± 0.1**	**1.4 ± 0,1**	2.6	0.5	2.8	1.6	3.0
Stressed OCR/ECAR	**0.7 ± 0.1**	**1.5 ± 0.1**	2.7	0.4	2.7	2.0	3.2

Depicted are the mean results ± standard deviation (*n* = 5 for P1, *n* = 6 for P2), except for the normalization parameters for which the mean value that was used for normalization is depicted. Results outside of the corresponding reference intervals are indicated in bold. CSU = citrate synthase units, SRC = spare respiratory capacity, OCR = oxygen consumption rate, ECAR = extracellular acidification rate. P5 and P95 represent the 5% percentile and 95% percentile of the results of the control samples.

## Data Availability

The data presented in this study are available on request from the corresponding author. The data are not publicly available due to pseudonymization issues.

## Supplementary Materials

The following supporting information can be downloaded at: www.mdpi.com/article/10.3390/cells11030489/s1, Table S1: Overview of cases with a NC_012920.1:m.9035T>C variant previously published in literature.
