# Supplementary material for: Clinical Heterogeneity in MT-ATP6 Pathogenic Variants: Same Genotype—Different Onset
Source: Cells. 2022 Jan 30;11(3):489. doi: 10.3390/cells11030489 (PMC8834419; doi:10.3390/cells11030489)
Supplement: Supplementary file 1 [file cells-11-00489-s001.zip › cells-1478134-supplementary.pdf]

Supplementary data

Table S1: Overview of cases with a NC_012920.1:m.9035T>C variant previously published in literature.

	# (pedigree)	Sex	Age at onset	Clinical findings	Biochemical findings				% Hetero-plasmy
					Muscle biopsy	Complex I, II, III, IV	Complex V	Other	
Sikorska *et al.*	16 (1), across four generations	8F<br>8M	Early to late childhood	Developmental delay, learning disability, progressive ataxia, dysarthria, sensory neuropathy	Normal 2/2	Normal 2/2	Decreased activity	Transmitochondrial cybrids showed a decreased basal ATP level and excessive ROS. The membrane potential was normal.<br>Elevated lactate ^{1}H-MRS<br>Resting serum lactate not elevated.	100 (blood)<br>8/8
Pfeffer *et al.*	4 (1) Three siblings and one of their sons (other children asymptomatic)	F	20y	Slurred speech, gait imbalance, cerebellar dysarthria, increased motor tone, brisk DTR, proximal weakness	N/A	N/A	N/A	N/A	N/A
		F	20y	Ataxia, brainstem syndrome, ophthalmoplegia, disordered eye movements, dysarthria, extensor plantar responses, absent DTR at ankles	Normal	N/A	N/A	N/A	96 (blood)
		F	16y	Leg cramps, gait ataxia, intermittent paresthesia, cerebellar dysarthria, leg spasticity, pyramidal weakness in legs, brisk DTR, extensor plantar reflexes.	N/A	N/A	N/A	N/A	90 (blood)<br>96 (muscle)
		M	2y	Speech delay, short stature	N/A	N/A	N/A	N/A	95 (blood)
Ng *et al.*	8 (3), no additional details provided	5F<br>3M	3y – 19y	Leigh syndrome (2/8), pathological brisk reflexes and/or positive Babinski sign (4/8), learning disability (5/7), dystonia (1/8), ataxia (8/8), neuropathy (3/7), pes cavus (2/3), retinitis pigmentosa (2/7)	N/A	N/A	N/A	N/A	> 90 (blood)<br>8/8
Rucheton *et al.*	1 (1)	N/A	<10y	Mental deficit, cerebellar syndrome, pyramidal syndrome, episodic paralysis, axonal sensory-	N/A	Normal	Normal activity in fibroblasts	Increased ratio of doubling time in glucose vs. galactose (fibroblasts)<br>MT-ATP6 subunit appeared decreased (muscle)	100 (muscle)<br>100 (fibroblasts)

				motor peripheral neuropathy			Decreased activity in muscle.		
Stendel *et al.*	3 (1), three siblings	M	Childhood	Multisystemic ataxia, neuropathy, learning disability	N/A	N/A	N/A	N/A	100 (n.a.)
		M	50	Multisystemic ataxia, neuropathy, dystonia, learning disability	Ragged red fibers	N/A	N/A	N/A	100 (n.a.)
		M	20	Multisystemic ataxia, neuropathy, dystonia	N/A	N/A	N/A	N/A	100 (n.a.)
Garret *et al.*	2 (1), mother and daughter	F	30	Learning disability, ataxia, axonal neuropathy, balance problems.	N/A	N/A	N/A	N/A	100 (urine)<br>near 100 (blood)
		F	53	Isolated axonal neuropathy, lower limb impairment	N/A	N/A	N/A	N/A	100 (urine)<br>near 100 (blood)
Haraux *et al.*	1 (1)	N/A	childhood	Psychomotor retardation, ataxia, sensorimotor peripheral neuropathy, cerebellar syndrome, pyramidal syndrome.	N/A	N/A	Decreased activity	Mild elevation of lactate level in blood and cerebrospinal fluid.	100 (blood, muscle)
P1	1 (1)	M	2y	Severe motor and speech delay, spastic ataxia without extra-neurologic involvement.	Normal	Normal	Normal	BN-page with in gel activity staining shows complex V subcomplexes<br>Reduced spare respiratory capacity.<br>Increase in extracellular acidification rate.	100 (blood)
P2	1 (1)	F	19y	Ataxia and ophthalmoplegia.. No cognitive or motor impairment.	Normal	Normal	Normal	BN-page with in gel activity staining shows complex V subcomplexes<br>Reduced spare respiratory capacity.<br>Increase in extracellular acidification rate.	100 (blood, fibroblasts, muscle)

N/A: no data available
